# Patients and agents – or why we need a different narrative: a philosophical analysis

**DOI:** 10.1186/s13010-018-0068-x

**Published:** 2018-10-14

**Authors:** Harald Walach, Michael Loughlin

**Affiliations:** 1 Department of Pediatric Gastroenterology, Medical University Poznan, Poznań, Poland; 2 Department of Psychology, University Witten-Herdecke, Witten, Germany; 3 Change Health Science Institute, Schönwalder Str. 17, 13347 Berlin, Germany; 4 University of West London, St Mary’s Rd, London, W5 5RF UK; 5 Nuffield Department of Surgical Sciences, University of Oxford, Oxford, UK; 6 England Centre for Practice Development, Canterbury, UK

**Keywords:** Agency, Causation, Context, Complexity, Lifestyle, Multifactoral diseases, Narratives, Patients, Person-centred, Philosophy of medicine

## Abstract

**Background:**

The success of medicine in the treatment of patients brings with it new challenges. More people live on to suffer from functional, chronic or multifactorial diseases, and this has led to calls for more complex analyses of the causal determinants of health and illness.

**Methods:**

Philosophical analysis of background assumptions of the current paradigmatic model.

**Results:**

While these factors do not require a radical paradigm shift, they do give us cause to develop a new narrative, to add to existing narratives that frame our thinking about medical care. In this paper we argue that the increased focus on lifestyle and shared decision making requires a new narrative of agency, to supplement the narrative of “the patient”. This narrative is conceptually linked to the developing philosophy of person-centred care.

**Conclusions:**

If patients are seen also as “agents” this will result in a substantial shift in practical decisions: The development and adoption of this narrative will help practitioners work with patients to their mutual benefit, harnessing the patients’ motivation, shifting the focus from treatment to prevention and preventing unnecessary and harmful treatments that can come out of our preoccupation with the patient narrative. It will also help to shift research efforts, conceptual and empirical, from “treating” and “battling” diseases and their purported “mechanisms” to understanding complex contributing factors and their interplay.

## Background

### The story of the “patient”

The stories we tell ourselves and others, and the words we use for this storytelling, are powerful [1, 2]. We know very well the story of the “patient”. It is, roughly, this: Some obnoxious thing from the outside, an invasive agent like a germ, an accident or injury, or some unknown inner cause like a hormonal dysfunction has caused a disease and transformed a healthy person into a patient. As a patient, this person is now a recipient of medical help. He or she is a sufferer in various aspects: He or she suffers from the disease – hence is a “patient”, from the Latin passive tense “pati – to suffer”, and “patiens – suffering”. The disease happens to him or her. And there is the real suffering from symptoms, for instance in an injury such as a burn. But he or she is also suffering, or allowing, the treatment, which is done to him or her by the medical profession, and, being a good patient, he or she is patient enough to allow the treatment to work – take the pills, do the prescribed exercises, wait, or allow the surgery, etc. This story of the patient has been the legitimizing story of medicine for a long time, and it is surely the legitimizing narrative for all acute and emergency cases, for relieving a patient from suffering in the strict sense of the word. It is the narrative of the treatment of emergencies and acute diseases and as such has done a good job.

However, this narrative has grown outdated, at least for all situations that are not emergencies. It’s not that it has become wrong or useless. Rather, its success has produced a new scenario which is in need of a new story, a new narrative, to complement the patient narrative. Now it is time for agents, or more precisely for turning patients into agents. We are using “agent” here in the philosophical sense of someone who is capable of action and activity [3]. This includes making choices and being aware of this capacity on part of the agent, and being able to harness this capacity on part of the medical profession, such as when a GP is suggesting to a patient that she should quit smoking or that she should drink less alcohol, and perhaps join an appropriate therapy or self-help group. She would not be able to do this, were she and her “patient” not implicitly considering the “patient” as “agent” already. We are highlighting this implicit understanding and are making a case for a better usage of “agency”, both in the theoretical-scientific sense and in the practical field. This new story is applicable, precisely because the old story was so successful. Where many patients died in previous times, they now live, thanks to this old patient narrative and thanks to the efforts of medicine. Where preterm infants had a very slim chance of surviving 30 years ago, they now do to become patients later on [4]. Where approximately 50 million sufferers of an influenza epidemic died 1918–1920 [5, 6], we have much fewer casualties due to infectious diseases in the West, because of the success of our narrative that recommends hygiene and immunization, and likely, because this narrative is embedded in the larger narrative of social, political and economic progress [7].

This means: more people live on to suffer from diseases, often multifactorial ones. This term is used to disguise our inability to make out one single culprit as a cause that could be combated, as the narrative requires. So we normally *choose* one culprit out of an array, usually the one that is supposed to be treatable most easily using the narrative of acute and emergency medicine. Take back pain as an example. Many people, patients and doctors, assume that at some point people with sedentary life-styles will get back pain or some other form of pain syndrome. Patients expect that this should be treatable by medications called “pain killers” for the very reason that they are supposed to treat pain. The standard narrative of emergency medicine is in operation and patients get treatment or treat themselves, as patients, as passive recipients of disease and treatment.

### The new narrative of agents – Why we need it

The new narrative of the agent tells a different story. It tells a story of an active agent who is spending way too much time indoors with too little movement, is therefore lacking essential stimuli and exercise, while at the same time increasing tension and a pro-inflammatory immunological situation through psychological stress, lack of sleep, lack of natural antioxidants in the food and increasing oxidative stress. While the standard narrative of emergency and acute medicine is likely to pick out one “major” culprit, such as inflammation by prescribing or buying pain killers, or bad posture and suggesting some back training and so forth, the new narrative of the agent is suggesting a different approach. Because back pain is not a uni-causal “disease” but rather a multifactorial event, where only the end result looks like an acute, causal disease, it is a matter of choice and depends on the narrative which one of these many “causes” we pick. If the disease is acute and threatening, the standard narrative might be indeed applicable. But if the aim is to change the propensity for suffering pain, clearly a different approach is needed. This is the new narrative of the agent. This views the “patient” as an “agent” who makes choices, behaves in a certain way and thereby contributes to, but is also able to change, conditions that lead to the susceptibility.

Note that the end product, the acute episode, will still leave the “agent” in a state of “patient”, but everything leading up to it might appear in a different light, if we look at the person in terms of an “agent”: the lifestyle choices he or she makes, whether to walk the stairs or take the lift, whether to eat fresh and quiet, or fast and junk food in a noisy environment, whether staying in front of a computer screen indoors all day, or using the breaks to sit outside in the sun, doing some exercise or taking the bike for the shopping, or addressing the friction in the relationship that may add to the psychological stress etc. The “agent” is someone who is active and is taking active part in his or her “treatment”. One aspect is shared decision making, which is part of evidence based medicine and a good example of the new narrative emerging [4]. But there is more to it. The “agent” is also someone who is, ultimately, responsible for his or her life in the sense that medical outcomes and the development of diseases depend, at least in part, on lifestyle choices taken earlier in life.

Crucially, the agent makes these choices in a social context, that may either enable or inhibit the agent’s capacity to make the choices that can contribute to improving her situation in the future, such that a full assessment of “the problem” and how best to address it will involve moving beyond the search for medical causes, to a consideration of the potentially unique circumstances of the suffering individual. [8] This may also involve discussing with the agent what counts as the best situation *for her*, as different agents may have different values and priorities, and no amount of medical expertise can make us experts on which trade-offs (for instance, of risks and benefits in the context of specific life choices) are the right ones for this person to make in the context of her particular life goals. (So, a ballet dancer suffering with asthma may resist an aggressive steroid therapy to control the wheezing, if she believes that the steroids may cause muscle weakness and fluid accumulation, thus causing her dancing to deteriorate. We can advise her of the options available and associated risks, but we are in no position to tell her she is simply wrong to prioritise maintaining the standard of her dancing over “bringing the disease under complete control.”) [9].

Thus, the narrative of “agency”, as we are using the term here, is an integral component of the emerging philosophy of “person-centred” care – an approach to healthcare influenced by philosophical work on personalism, emphasizing the “agency” and “inter-subjectivity” of all parties to the clinical encounter [8–12]. Increasingly the language of “person-centred health and social care” is being used to emphasise the need to focus on the whole person, whose biological and social aspects cannot be understood in isolation from each other, but must be conceived as integral features of the activity of living a whole life [8] – a philosophy emphasised not only in academic work on health philosophy but, increasingly, acknowledged as a much needed shift in thinking and practice in health policy documents [13–15].

The top three killers, heart disease, cancer and side effects of drugs [16], are dependent on life-style choices at least to a degree. For instance, combining four healthy lifestyle factors can reduce premature mortality from some of those diseases by 66%, in some even by 90% [17, 18].

This new narrative of the “agent” is not meant to replace the old narrative of the patient. It is meant to complement it. As persons we have many aspects. In some respects we are agents, in others patients. As Aristotle understood, these aspects are not dichotomous, and neither our agency nor our patient-hood can be understood in isolation from each other or from our social environment, as both are relational – we act and are acted upon within a social context [19, 20]. Not only is man a “social animal”, but agency (and subjectivity more broadly, including perception) are relational aspects or properties of the human animal [19–21].

So the “agent” in the agent narrative is any one of us, insofar as we aspire to increase our control of our lives, and our lived environment, for our own long-term benefit. The narrative of agency is applicable especially in those situations where the problem is functional, chronic or directly dependent on lifestyle, which will mean in probably 70% or so of the case load in general practice [22, 23].

### Obstructing habits and the power of collective practice

What does this new approach do for us? The new narrative points to ways where patients can become agents and do something for themselves, whether it is some form of self-care, or making different lifestyle choices, or taking responsibility for a good recovery, which sometimes also means compliance with prescribed treatments. It acknowledges patients’ agency. This agency we neglect at our own peril. Patients remain agents up to their last breath. They can resist or sabotage necessary treatments, but they very often also know when a treatment does them more harm than good. And agency can be harnessed. A type 1 diabetes patient who understands about diabetes, its consequences and danger, and who is treated as and sees himself or herself as agent, will be more proactive in regulating blood sugar. Someone at risk of getting diabetes type 2 can only be “treated” professionally well by addressing his or her agency, helping him or her to find a better nutritional pattern, taking time to find out about the nutritional habits, seeking out palatable alternatives and harnessing the power of agency [24].

The major driver of the pervasive force of the narrative of the patient is the availability of quick fixes. These are mostly surgery and emergency measures in case of accidents and emergencies, and often medications in case of other medical problems. The upside is: it works well in acute cases and emergencies. The downside is: it works less well in chronic and functional problems and it perpetuates the mythology of the patient in need of treatment. This driver is powerful, because it is connected to and dependent on the pharmaceutical industry and the health sector at large, which makes up a large proportion of the GDP in any developed economy. In Germany, for instance, the health sector, including the pharmaceutical industry, is the single largest industry [25, 26]. Therefore, it will be very difficult to change the narrative. Changing it, though, is important, even for our economies. Upholding the current patient narrative for chronic, functional and lifestyle dependent problems is foolish. It creates more problems, in the long run, than it solves. It keeps patients in an immature mode, making them recipients of treatment instead of agents that take care of themselves. Side effects of medications taken over a long period of time amass and create new “diseases” as side effects [27], apart from the fact that we have not enough data to support such prescriptions in most cases [28]. And it prevents the change to a different narrative that has a chance of turning the tide for chronic and functional problems, namely a focus on lifestyle, nutrition and simple actions.

To go back to our example of back pain: Of course one can use the patient narrative and keep taking NSAIDs or see an acupuncturist or physiotherapist, as many people do. This will likely reduce symptom load a bit, and the costs are massive: NSAIDs are the class of drugs with the single most deaths related to side effects [16, 29] and the economic burden of back pain is huge [22]. As an alternative one can switch to the agent narrative: One might then reconsider lifestyle choices, perhaps include some regular exercise into one’s life, or spending more time outdoors walking, biking or just playing, eating healthier and sorting social problems at work or at home proactively. And this is only part of what one might want to consider. Note that there is no single best practice, because agency also implies individuality. Everyone needs to do his or her own assessment, or the doctor with them, in shared decision making.

### The benefit of the new narrative

The powerful change with the agent narrative occurs on various levels:

- It introduces new, and likely more important, aspects into patient care and shifts the focus from treatment to prevention. Lifestyle choices are for the long haul and no quick fixes.
- It harnesses the patients’ motivation, and turning patients into agents will bank on their agency and responsibility.
- It prevents a multitude of likely unnecessary and very often harmful treatments that more often than not come out of our preconception that the patient narrative is without alternatives, rather than positive evidence that they work well and without side effects.

It shifts research efforts, conceptual and empirical, from “treating” and “battling” diseases and their purported “mechanisms” to understanding complex contributing factors and their interplay, from basic to applied-clinical, from isolated clinical to public health sectors.

Table 1 presents some further examples that could easily be elaborated but serve here the purpose of illustration. (Other diseases could be used, but we include these examples to illustrate the implications of the new narrative for practice.)

**Table 1:** Examples of How Changing the Narrative Might Effect Practice: Therapeutic Options Derived From Various Narratives; Note: The Evidence Base on Agent Narrative Based Practices Is Small Due to a Lack of Interest

Disease	Patient Narrative	Agent Narrative
Cardiovascular disease	Take drugs, avoid saturated fat and salt; surgery to prevent stenosis	Reconsider lifestyle choices [18], individualized nutrition program [52–54], stress-reduction, meditation and mindfulness, exercise [55, 56], consider psychosocial aspects and personal relationships [57, 58]
Rheumatoid arthritis	Control inflammation, use disease modifying agents	Check on nutritional antigens, consider fasting and an individually adapted vegetarian diet [59–61]; consider checking omega-3 to omega-6 ratio and supplementing, if low to reduce inflammatory propensity [62, 63]
Attention Deficit & Hyperactivity Disorder	Give dopamine agonists like ritalin	Check out social environment, nutritional and lifestyle patterns like overstimulation (caffeine, TV, media); [64] consider mindfulness practice; [65] check on omega-3 to omega-6 balance and consider supplementing (to improve neuroplasticity and learning) [66]
Mild Cognitive Impairment	Watchful waiting, consider cholinesterase inhibitors, if deterioration is obvious	Prevent medicalisation [30], consider changing dietary pattern towards more ketogenic intake avoiding sugars and simple carbohydrates [37, 38, 53], seek out cognitive stimulation [67], exercise [33], going into the sun and/or supplementing vitamin D [44, 45, 68], enhance social participation, contacts and volunteer work in the community, accept aging as a natural process [30, 46]

Let us close with one pertinent example, Alzheimer’s disease and dementia.

The patient-narrative approach to dementia runs somewhat like this (We are using Alzheimer’s as an example; it can be adapted to many other diseases or other types of dementia; see Table 1) [30]: The true causes of Alzheimer’s are unknown, but occur likely earlier in life, and are probably associated with various genetic risk factors that cannot be tackled. But what can and should be tackled are the likely pathological causes, such as the decay of the cholinergic system, the preponderance of amyloid beta plaques, neurofibrillary tangles and phosphorylated tau. So let’s understand this pathology better in order to be able to fix it, for instance with an inoculation, or by blocking some receptor or enzyme pathways. This would be good for everybody: patients could be treated, there would be the magic bullet everyone is waiting for at the end of the tunnel, and it would be good for the industry, because there will be a new blockbuster drug worth multi-billions of dollars.

The problem with this narrative is: it is very likely the wrong approach.

This is a good example of how the wrong narrative diverts our resources to inefficient roads [30, 31]. There have been some 81 or so substances in the pipeline of the industry, only some four of which, dependent on the countries, have made it to market [32]. None of these medications changes the course of the disease middle or long-term. They all only alleviate symptoms, if they do anything at all, at very high cost and with considerable side effects [33]. The inoculation approaches have been halted [32]. They worked well in animals on a mechanistic level but without changing the clinical outcome [34]. Many hundreds of billions of dollars have been invested in development, with very little progress [30]. All Alzheimer societies still give out prizes for finding the magic drug, the new breakthrough, which is very unlikely to occur. This is, because the presuppositions are likely wrong.

The presuppositions of the agent-narrative of Alzheimer’s are different. Here we suppose that the causes are multifactorial and likely also part of the human condition of getting old, i.e. Alzheimer’s is is not primarily a medical disease but a complex human condition. Perhaps if we all were to live to see 200 years we’d be all demented? We do not know. But at any rate, brain aging is a natural process that likely will occur more quickly in some people than in others, and some parts of it may be actually slowed down or diverted by preventive lifestyle measures [35]. Do we know? Not directly, because there have been very few resources to study this approach, precisely because of the dominance of the patient-narrative. But there are some indirect pieces of evidence. Some variance might be explained by the increasing amount of mercury from industrial and dental sources to which our societies are exposed [36]. This leads to selenium scavenging, because selenium is used by the body to bind mercury into the inactive mercury selenite, and thus deprives the body of its selenium resources. Most antioxidative enzymes in the body and in the brain are selenium based. So this process triggers a long term selenium deficiency which at one point might derail into an inflammatory cascade. Then there is the potential insulin resistance of neurons that have been exposed to high levels of sugar for a long time and that might make them ill functioning [37, 38]. There is the public health issue of a long term and population wide change in the omega-3 to − 6 ratio due to the shift in the fat profiles in our food. That shift reduces the availability of omega-3, which is necessary for axonal growth, neuro-neogenesis and downregulation of inflammatory processes [39–43]. There are other factors such as a lack of vitamin D [44, 45], a lack of social connectivity [46] and resources, a lack of cognitive activity [47] and physical exercise [33], and very likely they all combine. And all of those factors feed into a preventive model of dementia that makes the future patient a present agent who can do something to increase his or her chance of living a healthier life for longer [35].

### A note of caution

There is no promise of perfect health, no promise of a ban on suffering, as seems to be the driver of the patient narrative, but but a focus on agency, empowerment, self-efficacy, and self-fulfillment is promising. Calls for a new narrative recognize that promise and are increasing. [2].

What are the dangers of the narrative of agency? When many hear “agency” and “responsibility”, they think “guilt”. Certainly the term “responsibility” has been used in a way that strongly suggests guilt or culpability in some political discussions of health, with commentators – including government spokespersons – implying that patients whose “lifestyle choices” are causally responsible for their current medical conditions should be “de-prioritized” when decisions are made about making drugs and other forms of publicly funded medical treatment available [48–50].

While there may be economic or political agendas which make it attractive for some to link the promotion of “personal responsibility” for one’s health to the withdrawal of medical treatment for those deemed to have made “risky” or otherwise “unhealthy” choices, there is no logical link between the call for a new narrative of agency and such punitive measures. To treat someone as an agent is to attempt to understand the reasons for their actions in the context of their whole lives [51], with the goal of supporting them in making decisions that they will find benefit them in the future. Efforts to single out whole groups of patients (be they “smokers”, “drug addicts” or “the overweight”) for “de-prioritization” based on their supposedly irresponsible choices, oversimplify the complexity, diversity and individuality of human lives and, as such, are insensitive to the context-specific nature of rational, responsible choice that we have emphasized in this paper. The issues regarding the relationship between social factors, rationality and individual choice in context are complex. A choice that is risky in some respect might well be rational and right in other situations [50]. There is no logical or scientific basis for refusing to treat sports injuries of high risk sports on the grounds that the injured sportsperson “knew the risks” associated with an activity that also provided perceptible health benefits. Nor is there any simple, logical move from the ascription of causal to moral responsibility for a medical condition [42, 43]. (If there were, then the sportsperson who did indeed “know the risks” of sporting activity would automatically be held morally accountable for injuries arising in the course of activities that most credible health professionals would positively recommend. Such a view is patently indefensible.) The narrative of agency is a means of helping people avoid becoming patients, not an ad hoc rationale for penalizing those who are patients, as we will all be, at some point.

Our argument has been based on the evidence that a new narrative of agency is needed and beneficial. Our plea is that changing part of the narrative from patient to agent will be to the benefit of patients, or rather, agents, and doctors, as well as society. So let us gently convert patients into agents.
